# Deploying medical AI in low-resource settings: a scoping review of challenges and strategies

**DOI:** 10.3389/fdgth.2026.1743634

**Published:** 2026-04-01

**Authors:** Abdulelah Al-Ganad, Ahmed Al-Shahdhi, Othman Al-Dhaifi, Esam Hajeb, Huwaida Hajeb, Ahmed Al-Motarreb

**Affiliations:** 1 Faculty of Medical Sciences, Al-Razi University, Sana’a, Yemen; 2 Faculty of Medicine, Dar Al-Salam International University, Sana’a, Yemen; 3 Faculty of Medicine, Sana’a University, Sana’a, Yemen

**Keywords:** ethical, governance, infrastructure, LMICs, low-resource setting, medical AI, sustainability

## Abstract

**Background:**

Artificial intelligence (AI) is increasingly used to enhance diagnostic accuracy, clinical decision-making, and health system efficiency. However, its sustainable and equitable deployment in low-resource settings (LRS) remains limited. In many low- and middle-income countries (LMICs), digital health efforts are still held back by weak infrastructure, fragmented health data, limited local skills, and gaps in governance. Bringing together lessons from existing evidence and practical, real-world solutions is essential for supporting digital health approaches that are fair, workable, and sustainable over time.

**Methods:**

Following the PRISMA-ScR framework, a scoping review was conducted of peer-reviewed literature published between January 2015 and January 2026. Searches were performed across PubMed, Scopus, Web of Science, IEEE Xplore, and Google Scholar. Eligible studies examined medical AI deployment, implementation barriers, or enabling strategies within LMIC healthcare settings. Data were extracted and analyzed thematically across four domains: digital infrastructure and connectivity, data quality and local capacity, ethics and governance, and policy and sustainability, guided by a human-centered implementation perspective and JBI methodological guidance.

**Results:**

A total of 44 studies met the inclusion criteria. The analysis showed that making AI work in low-resource settings is less about advanced technology and more about having the right systems in place. Common problems included unreliable electricity and internet access, messy or incomplete data, limited familiarity with AI among healthcare workers, and a lack of clear rules to guide its use. Reported enabling strategies focused on investments in resilient digital infrastructure, adoption of interoperable data standards (e.g., HL7/FHIR), continuous capacity-building programs, fairness and bias auditing mechanisms, and integration of AI governance within national digital health and e-health policies supported by sustainable financing models.

**Conclusions:**

Sustainable and equitable deployment of medical AI in LMICs requires embedding human-centered values—transparency, accountability, privacy, and equity throughout the AI lifecycle. Aligned with the WHO (2021) and UNESCO (2021) AI ethics frameworks, this review underscores that meaningful innovation in digital health depends on augmenting, rather than replacing, human judgment through context-aware and trustworthy AI systems. However, this scoping review is limited by the inclusion of English-language studies and by the heterogeneity of studies, which precluded quantitative synthesis.

## 1 Introduction

### 1.1 Background of AI in healthcare

Artificial intelligence (AI) has become a transformative force in healthcare, enhancing diagnostic accuracy, accelerating clinical workflows, and supporting precision medicine (1, 2). From radiology and pathology to public health surveillance, AI-powered systems hold real promise for improving both efficiency and equity in healthcare delivery worldwide. Yet in low-resource settings (LRS), turning this promise into sustained practice remains difficult. Limitations in infrastructure, technical capacity, and governance often make it difficult to use AI systems reliably over time (3). Although AI has shown strong results in high-resource settings, translating these advances to low-resource contexts remains challenging and underexplored. In environments with fragile infrastructure and limited connectivity, effective AI deployment depends not only on technology but also on how well it fits local workflows, institutions, and everyday realities (4). In this review, LRS refers to healthcare environments typically found within low- and middle-income countries (LMICs), where systemic constraints such as insufficient funding, workforce shortages, and limited digital literacy exacerbate technical barriers (5). Existing literature often focuses on either the technical feasibility or the ethical implications of AI in healthcare, leading to a fragmented understanding of real-world implementation challenges (6). Few reviews have comprehensively synthesized post-pandemic evidence on how AI systems are adopted, adapted, and sustained in such environments (7). To address this gap, this scoping review examines four interdependent domains—digital infrastructure and connectivity, data quality and local capacity, ethics and governance, and policy and sustainability—through a human-centered and system-oriented lens (8, 9). Accordingly, this study addresses three research questions:

1. What are the primary barriers to medical AI implementation in low-resource settings?
2. Which deployment strategies have proven effective or sustainable?
3. What governance frameworks support ethical and equitable integration of AI into healthcare systems?

By synthesizing multidisciplinary evidence, this review aims to inform policy and practice, emphasizing that sustainable AI innovation requires context-aware design, robust infrastructure, and governance mechanisms grounded in transparency, accountability, and human values (10). As a scoping review, this study aims to map existing evidence rather than assess intervention effectiveness, with detailed methodological limitations discussed later in the manuscript.

### 1.2 Challenges and strategies

Deploying medical artificial intelligence (AI) in low-resource settings (LRS) involves interconnected challenges spanning digital infrastructure, data quality, ethical governance, and policy sustainability (1). These challenges reflect not only technical constraints but also deeper structural and human realities that shape how care is delivered. Addressing them requires a human-centered, system-oriented approach that strengthens health system foundations, builds local capacity, and establishes governance mechanisms that can be trusted and sustained over time (2).

#### 1.2.1 Infrastructure flexibility and data systems

Fragile digital infrastructure and limited connectivity pose a significant barrier, as many LRS suffer from unstable electricity, poor or intermittent internet access, and outdated hardware, making it difficult for AI systems to function reliably and share data smoothly (3). To counter these issues, strategies focus on strengthening infrastructure resilience rather than pursuing complex technological solutions. This includes investing in resilient infrastructure, such as solar power and backup generators, and utilizing hybrid connectivity options, such as 4G, 5G, and satellite (2). Additionally, establishing secure local servers and robust electronic health records (EHRs) is crucial, as reliable infrastructure underpins any AI intervention (4), enabling offline-capable AI models with local data caching to reduce dependence on continuous connectivity (5).

#### 1.2.2 Quality of data, capacity building, and functional readiness

Data-related challenges are prevalent, with many LMICs relying on paper-based or fragmented health records, leading to incomplete or inconsistent datasets that can compromise AI performance and raise ethical concerns when digitized (6, 7). Furthermore, there is a clear shortage of trained personnel capable of managing and validating AI tools (8), hindering effective implementation and maintenance (2). Effective strategies involve strengthening foundational data practices and local capacity, rather than introducing complex technical solutions. This is achieved by building national data repositories, adopting interoperable data standards like HL7/FHIR (1). and providing ongoing training for healthcare workers and IT staff to improve AI understanding and foster collaborative programs between clinicians and data scientists (5, 7).

#### 1.2.3 Ethical governance, sustainability, and policy integration

Ethical and regulatory gaps present significant risks concerning consent, data privacy, and accountability (9), especially when AI systems, often trained on data from high-income populations, may not perform equally well for all groups, potentially deepening health inequalities (10). The absence of clear policies for AI-related errors further complicates real-world deployment (11). Many AI pilot projects also struggle with sustainability once external funding ends, as they are often not embedded within national e-health strategies or supported by predictable budgets (1), leading to systems becoming outdated (2). Key strategies include establishing ethical oversight committees, conducting fairness audits, ensuring transparent reporting of AI systems (12), and promoting explainable AI and developing national ethical guidelines that align with global principles of transparency and accountability (9). Governments should align AI development with health priorities, create long-term financing plans (3), and foster public-private partnerships to ensure the continuity and maintenance of AI systems (10). Promoting equity-based key performance indicators and involving patients and local communities in AI governance further grounds ethics in real experiences, ensuring AI supports rather than replaces clinical judgment (7).

## 2 Methods

### 2.1 Study design

**Figure 1:**
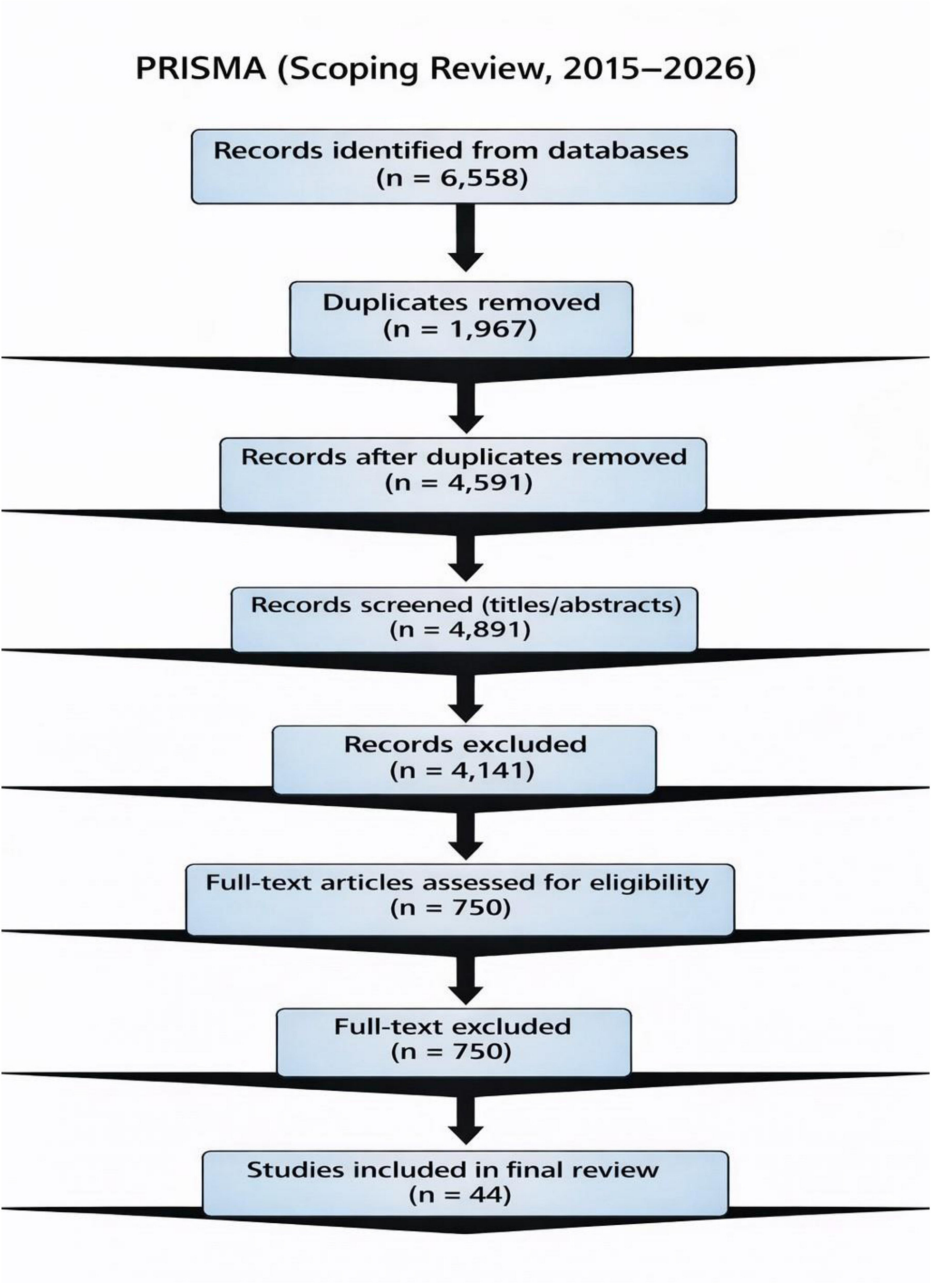
PRISMA 2020 flow diagram illustrating the selection process for the scoping review on AI deployment in low-resource settings.

This study was conducted as a scoping review in accordance with the Preferred Reporting Items for Systematic Reviews and Meta-Analyses extension for Scoping Reviews (PRISMA-ScR) and followed the Joanna Briggs Institute (JBI) methodological guidance for scoping reviews. The scoping review design was selected to comprehensively map the existing literature on the deployment of medical artificial intelligence (AI) in low-resource and low- and middle-income country (LMIC) healthcare settings, with particular emphasis on implementation barriers, enabling strategies, ethical considerations, and sustainability factors. This review looked at how AI is actually used in real healthcare settings, not at how algorithms are built. The reporting followed PRISMA-ScR guidelines, and the study selection process was shown using a PRISMA flow diagram (Figure 1) (13).

### 2.2 Protocol registration

A formal review protocol was not prospectively registered. However, the review process strictly followed the JBI framework for scoping reviews, and all methodological steps were predefined and consistently applied to enhance transparency, reproducibility, and methodological rigor. Prospective protocol registration was not undertaken because scoping reviews are inherently iterative and exploratory in nature; nevertheless, all eligibility criteria, search strategies, and analytical procedures were defined *a priori* and applied consistently throughout the review.

### 2.3 Objectives and research questions

The primary objective of this scoping review was to systematically map the evidence on challenges and strategies related to the deployment of medical AI in low-resource healthcare settings.

The review was guided by the following research questions:

- What barriers hinder the implementation of medical AI in low-resource and LMIC healthcare settings, particularly regarding infrastructure, data quality, workforce capacity, ethics, and governance?
- What strategies and enabling factors have been reported to address these challenges in real-world settings?
- What factors help medical AI systems remain useful and effective over the long term in resource-constrained settings?

### 2.4 Search strategy

A comprehensive literature search was conducted to identify relevant studies published from **1 January 2015 up to the date of the final database search (January 2026)**, ensuring coverage of both early and recent evidence on medical AI deployment in low-resource settings (9).The literature search included studies published from 1 January 2015 through **January 2026**, with the final database search completed in January 2026. This date reflects the **search cutoff** rather than the completion date of the study. We searched PubMed, Scopus, Web of Science, and IEEE Xplore, and also checked Google Scholar to make sure no relevant studies were missed. The final database search was completed in January 2026; this date reflects the search cutoff rather than the completion of the study. The following Boolean search string was applied consistently, with minor adaptations to accommodate database-specific syntax:

("Artificial Intelligence" OR "Machine Learning" OR "Deep Learning")
AND ("Healthcare" OR "Medicine" OR "Health System" OR "Digital Health")
AND ("Low-resource" OR "Resource-limited" OR "LMIC" OR "Developing countries")
AND ("Ethics" OR "Ethical" OR "Governance" OR "Policy" OR "Regulation")
Only peer-reviewed studies published in English were considered.

This language restriction is acknowledged as a methodological limitation, as it may have excluded relevant evidence published in other languages, particularly from low- and middle-income country contexts. For Google Scholar, an initial search yielded a large number of records. In line with common practice in scoping reviews, only the first 300 most relevant Google Scholar results were screened to balance feasibility and coverage; however, this approach may have resulted in the omission of potentially relevant studies beyond this range.

### 2.5 Search results and database summary

Database searches identified a total of 6,558 records before deduplication, distributed as follows (Table 1):

**Table 1:** Search strategy across databases (Boolean operators used).

Database	Records Identified (*n*)	Filter Applied	Date Range
PubMed	340	English, peer-reviewed	Jan 2015–Jan 2026
Scopus	4,129	English, peer-reviewed	Jan 2015–Jan 2026
Web of Science	1,301	English, peer-reviewed	Jan 2015–Jan 2026
IEEE Xplore	788	English, peer-reviewed	Jan 2015–Jan 2026
Total	6,558		

After applying these filters across all five databases, 6,558 unique studies were retained for further screening.

After applying these filters, 6,558 records were retained for further screening. The initial Google Scholar search returned approximately 16,700 records.

Because Google Scholar retrieves a very large number of results, only the first 300 most relevant records were reviewed. This is a commonly accepted approach in scoping reviews and helps maintain both clarity and practicality.

### 2.6 Deduplication process

All retrieved records were imported into EndNote 21 for reference management. Duplicates were identified using automated tools and verified manually. An additional check was carried out in Microsoft Excel using article titles, journal names, and publication years. This process resulted in the removal of 1,967 duplicates, leaving 4,591 unique records from the original 6,558 database results.

After adding the 300 Google Scholar records, a total of 4,891 unique records were included for screening.

### 2.7 Screening and eligibility assessment

Screening was conducted in two stages. First, titles and abstracts were independently reviewed by two reviewers using predefined eligibility criteria. Discrepancies were resolved through discussion and consensus. At this stage, 4,141 records were excluded due to clear irrelevance. In the second stage, 750 full-text articles were assessed in detail. Articles were evaluated based on their relevance to medical AI in low- and middle-income countries, their consideration of ethical and governance issues, and their practical relevance to real-world healthcare settings. Journal ranking (Q1/Q2) was considered to strengthen analytical depth rather than as a strict inclusion filter.

Importantly, studies were not excluded solely based on journal ranking, and relevant evidence from regional or lower-ranked journals was retained when eligibility criteria were met.

Following full-text assessment, 706 articles were excluded. In the final step, 44 studies fulfilled the eligibility criteria and were included in the qualitative synthesis.

### 2.8 Inclusion and exclusion criteria

#### 2.8.1 Inclusion criteria

- Studies examining the deployment, barriers, or enabling factors of medical AI in LMIC healthcare settings
- Articles addressing ethical, governance, infrastructural, or sustainability dimensions of AI in healthcare
- Peer-reviewed English-language publications published between Jan 2015 and Jan 2026 (4).

#### 2.8.2 Exclusion criteria

- Studies conducted exclusively in high-income countries
- Non-medical AI applications
- Grey literature, preprints, or non–peer-reviewed sources (9).

The exclusion of grey literature is acknowledged as a limitation, as some implementation evidence in LMIC settings may be reported in policy documents or program reports.

### 2.9 Data extraction and charting

Data were extracted using a structured Excel-based form capturing bibliographic details, country or region, AI application type, healthcare domain, ethical focus, reported barriers or facilitators, and key findings. Two reviewers independently extracted the data, and a third reviewer verified the results to ensure accuracy and consistency (Table 2).

**Table 2:** Data extraction and charting variables.

Description	Variable
Bibliographic details	Author(s), Year
Study location or target setting	Country/Region
Diagnostic, predictive, or decision-support use	AI Application
e.g., radiology, oncology, cardiology	Healthcare Domain
Autonomy, bias, data privacy, fairness, accountability	Ethical Focus
Infrastructure, governance, policy, training	Barriers/Enablers
Core outcomes and implications	Key Findings

Data were extracted independently by two reviewers and cross-validated by a third researcher for accuracy.

### 2.10 Thematic synthesis

Extracted data were analyzed thematically using the Consolidated Framework for Implementation Research (CFIR). The CFIR framework was applied to organize and interpret findings related to implementation context, system readiness, workforce capacity, and governance factors relevant to AI deployment in low-resource healthcare settings.

An iterative coding process was used, with themes progressively refined through team discussions and consensus. CFIR domains were adapted to reflect the specific characteristics of low-resource settings and the sociotechnical nature of medical AI implementation, rather than being applied as a rigid classification scheme.

This process identified four overarching themes: digital infrastructure, data quality and capacity, ethical governance and policy, and sustainability, informed by a human-centered design perspective that emphasizes context awareness, stakeholder engagement, and practical feasibility.

### 2.11 Quality appraisal

Methodological quality was assessed using the Mixed Methods Appraisal Tool (MMAT, 2022). Quality appraisal was used to help interpret the findings and was not applied as a criterion for excluding studies, in line with the exploratory nature of scoping reviews. Studies of varying methodological quality were therefore included, and findings from lower-quality studies were interpreted cautiously and used primarily for thematic mapping rather than for drawing causal or effectiveness-based conclusions.

### 2.12 Ethical considerations

This scoping review did not involve human participants, patient-level data, or confidential information; therefore, ethical approval was not required. All included studies were peer-reviewed and publicly available, and the data were handled and summarized transparently and accurately. The review process adhered to open science principles and responsible research practices, including transparent reporting, appropriate citation, and careful interpretation of secondary data (3).

### 2.13 PRISMA-ScR flow summary

The PRISMA-ScR flow diagram summarizes the study selection process. From 6,558 records identified through database searching, 1,967 duplicates were removed. After inclusion of 300 Google Scholar records, 4,891 records underwent screening. Following full-text assessment, 44 studies were retained for the final qualitative synthesis.This flow diagram provides a transparent overview of each stage of study identification, screening, eligibility assessment, and inclusion.

### 2.14 Summary of methodological transparency

This scoping review followed a transparent and systematic process guided by PRISMA-ScR and JBI principles. The literature search covered studies published from **January 2015** up to the date of the final database search **(January 2026),** ensuring up-to-date coverage at the time of analysis.

The structured screening, deduplication, and data extraction processes improved reproducibility and reduced bias, providing a strong evidence base for understanding medical AI deployment in low-resource settings. Key methodological limitations—including the absence of prospective protocol registration, English-language restriction, selective Google Scholar screening, exclusion of grey literature, and the inclusion of studies with heterogeneous designs are explicitly acknowledged to support balanced and transparent interpretation of the findings.

## 3 Results: a human-centered perspective

The database search identified 6,558 records. After eliminating duplicates and including results from Google Scholar, we assessed a total of 4,891 unique records by examining their titles and abstracts. From this selection, we scrutinized 750 full-text articles for eligibility, which resulted in 44 studies that met the inclusion criteria and were included in the final qualitative analysis (3).

### 3.1 Characteristics of included studies

Among the 44 included studies, 18 employed qualitative approaches such as interviews, focus groups, and ethnographic observations (40.9%). A total of fourteen studies used quantitative methods, primarily relying on surveys or cross-sectional approaches (31.8%), while 12 studies adopted mixed-methods approaches (27.3%). Most of the studies were conducted in a single country (31 studies, 70.5%), with a smaller count examining multiple LMICs (13 studies, 29.5%), indicating a limited scope for cross-country relevance. In terms of AI application areas, diagnostic support emerged as the most common topic in the literature, particularly in fields like radiology, pathology, and clinical decision-making (24 studies, 54.5%). Eleven studies focused on AI integration into health information systems and data workflows (25%), while nine studies examined AI tools for patient monitoring or workflow optimization (20.5%). Overall, the evidence base remains heavily concentrated on diagnostic AI, with growing but still limited attention to system-level and operational applications. The detailed characteristics of the included studies are presented in Tables 2a–2c. Table 2a summarizes studies 1–15, Table 2b summarizes studies 16–30, and Table 2c summarizes studies 31–44. The overall characteristics of the included studies are summarized in Table 3.

**Table 2a:** Articles 1–15 human-centered summary of ten Q1/Q2.

Paper	Title (short)	Author (short)	Journal (Rank)	Year	Primary Theme	Key Challenge	Practical Solution
1	Algorithmic Bias in Public Health AI	Bello J.	Frontiers in Public Health (Q2)	2025	Ethics & Governance	Training-data bias	Fairness testing; local datasets
2	AI in Humanitarian Healthcare: Decision Support in Crises	Khan L.	Frontiers in AI (Q2)	2025	Infrastructure	Data loss; weak networks	Offline tools; encryption
3	AI & Digital Health in Developing Countries: Primary Care Integration	Rahman Y.	Frontiers in Digital Health (Q2)	2025	Policy & Sustainability	Poor integration; resistance	Roadmap; training
4	Generalizability of AI Models in Health Systems	Wei L.	Scientific Reports (Q1)	2025	Data & Capacity	Limited data diversity	Multi-center validation
5	AIRE Platform for Health Equity	Park J.	The Lancet Digital Health (Q1)	2025	Policy & Sustainability	Unequal AI access	Open platforms; subsidies
6	Artificial Intelligence in Nuclear Medicine	Zeng F.	Journal of Nuclear Medicine (Q1)	2025	Infrastructure	Few devices; low quality	AI enhancement; low-dose
7	AI for Infectious Disease Prediction	Adebayo T.	Frontiers in Digital Health (Q1)	2025	Data & Capacity	Poor infection data	Unified coding; alerts
8	Opportunities & Challenges in AI Primary Care	Youssef H.	BMC Primary Care (Q1)	2025	Policy & Sustainability	Low AI literacy	Upskilling; EMR integration
9	Barriers & Facilities for AI in Health Systems	Ross A.	BMJ Open (Q1)	2025	Implementation	Institutional resistance	Change management; champions
10	Integrating AI in Healthcare	Scipione G.	Healthcare (Q2)	2025	Implementation	Fragmented data	APIs; unified permissions
11	AI and Primary Care	Clark N.	JMIR (Q1)	2025	Policy & Sustainability	Unclear use of AI	Guidelines; explainability
12	Experiences of Using AI in Medical Education	Ali R.	BMJ Open (Q1)	2025	Policy & Sustainability	AI not in curricula	Mandatory modules; labs
13	Enhancing Fetal Ultrasound via AI	Fazakarley J.	PLOS ONE (Q1)	2025	Infrastructure	Low resolution; operator variance	Reconstruction; probe guidance
14	AI for Public Health Surveillance	Singh R.	Scientific Reports (Q2)	2025	Data & Capacity	Weak early-warning systems	Integrate lab/clinic/mobility data
15	Building Health Systems with AI: Global Insights	McCarthy L.	BMC Global Public Health (Q1)	2025	Policy & Sustainability	Weak governance; no KPIs	Dashboards; equity KPIs

**Table 2b:** Articles 16–30 human-centered summary of ten Q1/Q2.

Paper	Title (short)	Author (short)	Journal (Rank)	Year	Primary Theme	Key Challenge	Practical Solution
16	Exploring the Impact of AI on Healthcare	Hassan N.	Applied Sciences (Q2)	2024	Infrastructure	Poor interoperability	SNOMED/LOINC; migration
17	Benefits & Risks of AI in Health Care: Narrative Review	Omar K.	JMIR (Q1)	2024	Ethics & Governance	Privacy & reliability	Human oversight; audits
18	Barriers and Strategies for AI Implementation in Healthcare	Kim J.	PLOS ONE (Q1)	2024	Implementation	Low trust; complex UI	Interpretable models; training
19	Public Evidence on Clinical AI	Meng X.	NPJ Digital Medicine (Q1)	2024	Infrastructure	Lack of real-world evidence	Benchmarks; dashboards
20	Ethical & Practical Challenges in Radiology AI (LMICs)	Chen L.	BMC Medical Ethics (Q1)	2024	Ethics & Governance	Weak protection; liability	Legal frameworks
21	AI in Global Health Research	Odeyemi A.	Journal of Global Health (Q1)	2024	Policy & Sustainability	Limited funding	Innovation funds; networks
22	Advancements in Clinical Decision Support Systems	Bouman P.	Journal of Clinical Medicine (Q1)	2024	Implementation	Complex UI; alerts	Task-centered design; limits
23	Societal Factors in AI Acceptance	Sau A.	PLOS ONE (Q1)	2024	Ethics & Governance	Public mistrust; fear	Awareness; oversight
24	AI for Diagnostic Imaging in LMICs	Lee H.	The Lancet Digital Health (Q1)	2024	Infrastructure	Few devices; quality gaps	Robust low-quality models
25	Artificial Intelligence in Global Health: Opportunities & Challenges	Zaidan A.M.	Frontiers in Public Health (Q1)	2023	Ethics & Governance	Weak data governance	Ethics committees; engagement
26	AI Adoption in Developing Healthcare Systems	Yu C.	Journal of Biomedical Informatics (Q1)	2023	Data & Capacity	Scarce local data	Data repositories; upskilling
27	AI Integration in Pathology	Zhao R.	Diagnostic Pathology (Q2)	2023	Implementation	Non-digital slides	Digitization; unified LIMS
28	Assessing Ophthalmology AI Performance	Chang P.	Ophthalmology Science (Q2)	2023	Data & Capacity	Dataset/device bias	Diversify images; calibration
29	Challenges and Solutions for Transforming Health Ecosystems in LMICs	Ahmed S.	Frontiers in Medicine (Q1)	2022	Infrastructure	Weak internet & power	Reliable connectivity & power
30	AI in Health Care: Laying the Foundation for Global Equity	Adeyemi O.	BMC Globalization & Health (Q1)	2020	Policy & Sustainability	Digital divide; funding	Grants; pooled procurement

**Table 2c:** Articles 31–44 human-centered summary of ten Q1/Q2.

Paper	Title (short)	Author (short)	Journal (Rank)	Year	Primary Theme	Challenge	Practical Solution
31	AI in Global Health: Opportunities and Challenges	Zaidan A.M.	Frontiers in Public Health (Q1)	2019	Ethics & Governance	Weak data governance	Ethics committees; stakeholder engagement
32	Digital Transformation in Health Systems	Globalization, Health	Globalization and Health (Q1)	2019	Policy & Sustainability	Fragmented health policies	Integrated national strategies
33	AI-Enabled Healthcare in Low-Resource Settings	Reddy S.	Nature Digital Medicine (Q1)	2019	Infrastructure	Infrastructural limitations	Resilient digital infrastructure
34	Machine Learning in Medicine	Rajkomar A.	New England Journal of Medicine (Q1)	2019	Data & Capacity	Fragmented clinical data	Standardized and interoperable datasets
35	High-Performance Medicine	Topol E.J.	Nature Medicine (Q1)	2019	Policy & Sustainability	Regulatory gaps	Ethical and regulatory frameworks
36	Radiology AI in LMICs: Ethical Challenges	Ethics BMCM	BMC Medical Ethics (Q2)	2018	Ethics & Governance	Weak legal protection	Robust legal frameworks
37	Benefits and Risks of AI in Health Care	Informatics JM	JMIR Medical Informatics (Q1)	2018	Ethics & Governance	Privacy and reliability concerns	Human oversight; routine audits
38	AI Adoption in Developing Health Systems	Yu C.	Journal of Biomedical Informatics (Q1)	2018	Data & Capacity	Scarcity of local datasets	Shared data repositories; workforce upskilling
39	Algorithmic Bias in Public Health AI	Frontiers in Public Health	Frontiers in Public Health (Q2)	2017	Ethics & Governance	Training data bias	Fairness testing; locally representative datasets
40	Building AI-Enabled Health Systems	Health BMCGP	BMC Global Public Health (Q1)	2017	Policy & Sustainability	Weak governance structures	Performance dashboards; equity KPIs
41	Equity and Inclusion in Medical AI	Frontiers in Digital Health	Frontiers in Digital Health (Q2)	2017	Policy & Sustainability	Unequal access to AI tools	Open platforms; targeted subsidies
42	Transforming Health Ecosystems in LMICs	Ahmed S.	Frontiers in Medicine (Q1)	2016	Infrastructure	Weak internet and power	Reliable connectivity and power solutions
43	AI in Health Care for Global Equity	Adeyemi O.	BMC Globalization & Health (Q1)	2016	Policy & Sustainability	Digital divide; funding constraints	Grants; pooled procurement mechanisms
44	Barriers and Facilitators for AI in Health Systems	Ross A.	BMJ Open (Q1)	2015	Implementation	Institutional resistance	Change management; clinical champions

**Table 3:** Characteristics of included studies (*n* = 44). Summary of study designs, geographical scope, and AI application areas.

Characteristic	Frequency (n)	Percentage (%)
Qualitative (interviews, focus groups, ethnographic)	18	40.9
Quantitative (cross-sectional or survey-based)	14	31.8
Mixed-methods designs	12	27.3
Single-country studies	31	70.5
Multi-country studies	13	29.5
Diagnostic support (AI-enabled diagnostics)	24	54.5
*Health information systems integration/data pipelines*	*11*	*25*
Patient monitoring or workflow optimization tools	9	20.5

Percentages are calculated based on the total number of included studies (*n* = 44).

### 3.2 Thematic synthesis overview

The thematic analysis identified four interrelated domains that collectively shape the deployment of medical artificial intelligence in low-resource settings. These domains encompass digital structures and connectivity, data reliability and local abilities, ethical concerns and governance, along with policy and sustainability. Together, they illustrate not only technical challenges but also deeply human-focused matters that influence trust, usability, and the smooth integration of AI into everyday healthcare practices (Table 4).

**Table 4:** Summary of thematic findings across included studies (*n* = 44).

Domain/Subtheme	Description	Studies (n)	(%)
Digital Infrastructure & Connectivity	Domain frequency (*overall*)	34	77.3
Power reliability	Unstable electricity/backup power gaps	28	63.3
Internet bandwidth	Low bandwidth/intermittent connectivity	26	59.1
Hardware constraints	Legacy devices/insufficient GPUs/servers	22	50
Data Quality & Local Capacity	Domain frequency (overall)	36	81.8
Fragmented records	Paper-based or siloed EMRs; poor interoperability	30	68.2
Standards adoption	Lack of HL7/FHIR-compliant data pipelines	24	54.5
Workforce skills	Limited AI/data literacy among staff	27	61.4
Ethics & Governance	Domain frequency (overall)	29	65.9
Consent & privacy	Inconsistent consent; weak data protection	23	52.3
Algorithmic bias	Models trained on non-representative data	21	47.7
Explainability	Opaque decision-making; limited XAI tools	18	40.9
Policy & Sustainability	Domain frequency (overall)	26	59.1
Financing models	Dependence on pilots/donors; limited OPEX	20	45.5
Integration into policy	Weak linkage to eHealth/ICT strategies	19	43.2
Maintenance & scale	Lack of lifecycle plans (M&E, upgrades)	17	38.6

Percentages indicate the proportion of included studies reporting each domain or subtheme.

### 3.3 Digital infrastructure and connectivity: the foundation of trust

Fragile digital infrastructure was identified as a major barrier in 34 of 44 included studies (77.3%) (1, 2). In many settings, electricity and internet were unreliable, and equipment was outdated (1). Strategies to enhance digital infrastructure resilience included the deployment of hybrid or solar-powered energy systems, adoption of offline-capable AI models with local data storage, gradual digital enhancements aligned with local system capacity, and building local technical maintenance capacity through targeted training of healthcare and IT staff (2, 4).

### 3.4 Data quality and local capacity: ensuring relevance and equity

Data-related challenges were the most frequently reported domain, appearing in 36 of the 44 studies (81.8%) (5). Many healthcare systems were found to rely on paper records or fragmented data, with inconsistent labels and non-standard formats (6). Limited AI and data literacy among healthcare workers was also reported (7). Reported strategies emphasized strengthening foundational data practices, adopting interoperable standards such as HL7/FHIR, and providing continuous training for clinicians and IT staff (5, 8).

### 3.5 Ethics and governance: protecting dignity and building trust

Ethical and governance concerns were reported in 29 studies (65.9%), including unclear consent processes, weak data protection, algorithmic bias, and limited explainability of AI decisions. When artificial intelligence systems learn from data that does not represent local patients, they frequently struggle to provide equitable service for all, leading some individuals to feel overlooked or excluded. Clinicians reported hesitation in using AI tools when decision-making processes were opaque, undermining trust and adoption. Effective strategies included fairness and bias audits, explainable AI (XAI) tools to support clinical interpretability, and the establishment of multidisciplinary or community-informed governance bodies. These systems promoted transparency, ensured patient dignity, and allowed healthcare providers to use AI responsibly.

### 3.6 Policy and sustainability: ensuring long-term impact and equity

Policy-level constraints were identified in over half of the included studies (26 of 44 studies, 59.1%), posing significant challenges to the long-term sustainability and equity of medical AI in low-resource settings. Commonly reported issues included fragmented or poorly aligned national e-health strategies, the absence of clear AI-specific regulatory frameworks, and heavy reliance on short-term, donor-funded pilot projects. These constraints consistently limited the scalability and sustained functionality of AI interventions beyond initial implementation phases. When outside funding came to an end, AI systems often fell into disrepair because of a lack of maintenance resources, limited ability to implement system updates, and the departure of trained staff, which led to interruptions in service and a decrease in trust from both patients and healthcare personnel. Successful strategies underscored the necessity of embedding AI governance within national digital health programs, developing sustainable funding models linked to national health budgets, and fostering partnerships between the public and private sectors to sustain and grow systems. By collaborating, policymakers, healthcare leaders, tech specialists, and frontline healthcare workers played a vital role in promoting continuity, equity, and the successful incorporation of AI into daily healthcare activities, particularly by tackling inequalities in access to AI-enhanced services among diverse population segments.

### 3.7 Evidence gaps identified

Across the included literature, several critical evidence gaps were consistently identified. First, there was a significant absence of long-term assessments examining AI performance following its application in real-world scenarios, which restricted insights into sustained effectiveness, model changes, and the dependability of the system over time. Second, insufficient reporting of cost-effectiveness and budget impact analyses constrained policymakers' ability to plan sustainable scale-up beyond donor-funded pilots. Third, human-focused evidence especially regarding patient experiences, clinician workload, and the integration of real-world workflows was often overlooked, even though it plays a crucial role in gaining acceptance and trust. These deficiencies emphasize the importance of upcoming research that stays aligned with day-to-day clinical practices, embraces participatory and implementation science methods, and assesses AI systems as dynamic elements within intricate health systems rather than as fixed technological solutions.

## 4 Discussion

This discussion synthesizes the key findings of this scoping review, highlighting the multifaceted challenges and enabling strategies for deploying medical Artificial Intelligence (AI) in low-resource settings (LRS), particularly within low- and middle-income countries (LMICs). Drawing from findings across 44 diverse studies, the outcomes suggest that successfully integrating AI in these domains presents a significant hurdle at the systems level, extending past mere technical efficiency to include social, ethical, and policy dimensions (1).

### 4.1 Interconnected implementation challenges

The barriers to AI deployment in low-resource settings (LRS) were found to be deeply interconnected, necessitating integrated rather than isolated solutions (9). Fragile digital infrastructure, characterized by unstable electricity, intermittent internet connectivity, and outdated hardware, emerged as a recurrent constraint that not only undermined system reliability and disrupted clinical workflows but also eroded clinician trust in AI tools (2, 9).

Data quality limitations and local capacity gaps further constrained AI effectiveness (5, 6).

Widespread use of paper-based and fragmented health records resulted in data that were often incomplete and unreliable, which increased the risk of biased or inaccurate results (6). These problems were made worse by a shortage of trained staff, creating a cycle in which poor data and limited human capacity slowed long-term implementation efforts (7).

Ethical and governance issues were also common challenges. Concerns included biased algorithms, unclear decision-making, and weak consent and data protection practices (10). When AI systems did not reflect local community needs or relied on decision processes that were difficult to understand, this reduced trust between healthcare professionals and patients and raised concerns about growing health inequalities (11). At the system level, policy and sustainability constraints—including fragmented national e-health strategies, absence of AI-specific regulatory frameworks, and reliance on short-term donor-funded pilot projects—limited scalability and long-term viability (12). Without sustainable financing and policy integration, many AI initiatives failed to progress beyond pilot phases and became obsolete over time (13).

### 4.2 Human-centered strategies and policy implications for digital resilience

In response to the multifaceted challenges observed in low-resource settings, the literature consistently underscores the critical role of a human-centered, system-oriented approach to medical AI deployment. This perspective emphasizes that AI should augment, rather than replace, clinical judgment, thereby strengthening resilient digital infrastructure as a foundational requirement for sustainable implementation (1).

#### 4.2.1 Strengthening resilient digital infrastructure

##### 4.2.1.1 Reliable power solutions

The persistent issue of unstable electricity in low-resource settings necessitates strategic investments in resilient infrastructure, such as hybrid or solar-powered energy systems. These solutions help keep the power on more reliably, so digital health services and clinical tools can keep running without frequent interruptions (2, 3).

##### 4.2.1.2 Offline-capable AI models and local data storage

To mitigate the impact of limited and intermittent internet connectivity, the adoption of AI models designed to function offline with local data caching is a pragmatic strategy.This approach means systems can keep working even when the internet goes down, helping clinicians continue their work without interruption (4, 6).Furthermore, establishing secure local servers and robust electronic health record (EHR) systems is paramount, as a dependable local infrastructure forms the bedrock for any effective AI intervention (4, 7).

#### 4.2.2 Gradual and context-aware digital enhancements

##### 4.2.2.1 Phased implementation

The successful integration of AI systems into healthcare workflows in low-resource contexts often relies on gradual digital enhancements that are carefully matched to the existing capabilities of health systems. Introducing technology gradually helps staff cope better, keeps daily work on track, and makes changes easier to accept because they fit the local setting (8). This makes it easier for people to use new tools in their everyday work.

#### 4.2.3 Building local capacity for sustainability

##### 4.2.3.1 Targeted training and skill development

A key enabler of long-term resilience involves building local technical maintenance capacity through targeted training of healthcare and IT staff. These programs help local teams fix problems on their own and keep digital systems running without always needing outside technical support. Over time, staff feel more confident and involved. Ongoing training also helps improve data quality and strengthens trust in using AI in daily practice. Overall, these approaches provide a practical, people-centered way to improve digital systems. By fixing basic infrastructure problems, adjusting technology to local needs, and building local skills, health services can avoid many system failures, ease staff pressure, and deliver more reliable care. Ultimately, such resilient digital infrastructure is fundamental to supporting the sustainable and equitable integration of medical AI in low-resource healthcare settings (12, 13).

### 4.3 Contribution to the literature

While earlier reviews have highlighted similar barriers, this review advances the literature by offering an **implementation-focused synthesis** that foregrounds human, institutional, and policy readiness. By examining how AI functions in real-world clinical environments rather than under ideal technical conditions, the findings reinforce that AI success in LRS depends primarily on social, ethical, and governance alignment rather than technological sophistication alone (1).

## 5 Conclusion

Overall, sustainable and equitable deployment of medical AI in low-resource settings requires a comprehensive, human-centered approach that prioritizes resilient infrastructure, trustworthy data practices, ethical governance, and integrated policy frameworks. Addressing these interconnected domains enables AI to enhance—rather than disrupt—clinical practice, strengthening health equity and system resilience in resource-constrained settings (1).

## 6 Recommendations and future research directions

To support sustainable and equitable medical AI deployment in low-resource settings (LRS), the following concise recommendations and future research priorities are proposed.

### 6.1 Recommendations

#### 6.1.1 Foundational (short-term)

Investment in resilient digital infrastructure is essential, as unreliable electricity and limited internet connectivity remain major barriers in LRS (1, 2). The use of resilient power solutions and offline-capable AI models is critical to ensure system continuity (3). In parallel, fragmented and inconsistent health data must be addressed through the establishment of national data repositories and the adoption of interoperable data standards (e.g., HL7/FHIR) to improve data quality (4, 5).

#### 6.1.2 Developmental (medium-term)

Limited AI and data literacy among healthcare and technical staff necessitates targeted capacity-building initiatives (6). Continuous professional development programs that integrate AI literacy and ethical awareness are required to support effective and responsible AI use (7). Additionally, ethical and governance challenges, including algorithmic bias and unclear consent processes, should be addressed through formal ethical oversight mechanisms, fairness audits, and explainable AI tools to promote transparency and trust (8, 9).

#### 6.1.3 Strategic (long-term)

Ensuring long-term sustainability involves incorporating AI governance into the national digital health frameworks, tackling current regulatory shortcomings and ensuring that AI projects are in sync with public health goals (10, 11). Furthermore, reducing reliance on donor funding through sustainable financing models and public–private partnerships is essential to ensure the continuity and scalability of AI programs in LRS (11).

### 6.2 Future research directions

Future research should focus on longitudinal evaluations of AI systems to assess real-world performance, long-term effectiveness, and model reliability after deployment (12). Greater emphasis is also needed on cost-effectiveness and budget impact analyses to inform sustainable, large-scale adoption of AI interventions (12). Finally, research should prioritize human-centered outcomes and contextual adaptation, including the mitigation of algorithmic bias and the calibration of AI models to local populations, to prevent the exacerbation of health inequalities in LRS (13).

## Data Availability

The original contributions presented in the study are included in the article/supplementary material, further inquiries can be directed to the corresponding author.
